# Deep learning reconstruction-applied PROPELLER diffusion-weighted imaging in uterine malignancies: a comparison study with MUSE

**DOI:** 10.1007/s11604-026-02048-9

**Published:** 2026-07-15

**Authors:** Takuro Gonda, Ryoya Ochiai, Hiroto Yunaga, Kyohei Okuda, Yudai Ota, Shinya Fujii

**Affiliations:** 1 Division of Radiology, Department of Multidisciplinary Internal Medicine, Faculty of Medicine, Tottori University, 36-1, Nishi-Cho, Yonago, Tottori, 683-8504 Japan; 2 Department of Clinical Radiology, Tottori University Hospital, Yonago, Japan

**Keywords:** Magnetic resonance imaging, Diffusion-weighted imaging, Periodically Rotated Overlapping parallEL Lines with Enhanced Reconstruction (PROPELLER), MUltiplexed Sensitivity Encoding (MUSE), Deep learning, Uterine neoplasms

## Abstract

**Purpose:**

To compare the image quality of diffusion-weighted imaging (DWI) between deep learning reconstruction (DLR)-applied Periodically Rotated Overlapping ParallEL Lines with Enhanced Reconstruction (PROPELLER) and MUltiplexed Sensitivity Encoding (MUSE) sequences in MRI of uterine malignancies.

**Materials and methods:**

This retrospective study included 66 MRI examinations of 48 patients. The cohort consisted of cases with uterine malignancies (cervical cancer (n = 50), endometrial cancer (n = 15) and endometrial stromal sarcoma (n = 1)) at different clinical settings (initial staging, post-treatment, or recurrence). DWI was performed using both DLR-applied PROPELLER and MUSE sequences using b-values of 0 and 800 s/mm^2^. Two independent reviewers evaluated overall image quality, artifacts, sharpness, and lesion conspicuity using a 5-point Likert scale. Apparent diffusion coefficient (ADC), apparent signal-to-noise ratio (aSNR), and apparent contrast-to-noise ratio (aCNR) were quantitatively measured in both sequences.

**Results:**

In the qualitative assessment, DLR-applied PROPELLER-DWI showed significantly fewer artifacts and better lesion conspicuity than MUSE-DWI. No significant difference was found in overall image quality or sharpness. Inter-reader agreement was slight to moderate, except for strong agreement on DLR-applied PROPELLER-DWI lesion conspicuity (*κ* = 0.696, good). Quantitatively, the mean ADC values were significantly higher with DLR-applied PROPELLER-DWI compared to MUSE-DWI for both readers (*p* < 0.001). Furthermore, both aSNR and aCNR were significantly higher with DLR-applied PROPELLER-DWI compared to MUSE-DWI.

**Conclusions:**

DLR-applied PROPELLER-DWI demonstrated fewer artifacts and better lesion conspicuity than MUSE-DWI, with higher measured aSNR and aCNR. However, DLR-applied PROPELLER-DWI yielded significantly higher mean ADC values.

**Supplementary Information:**

The online version contains supplementary material available at https://doi.org/10.1007/s11604-026-02048-9.

## Introduction

Diffusion-weighted imaging (DWI) has been an indispensable MR sequence in routine gynecologic practice [1]. DWI complements the morphological information obtained from conventional MR sequences, such as T2-weighted imaging (T2WI). Furthermore, DWI provides functional information by deriving its image contrast from differences in the motion of water molecules between tissues. Generally, the motion of water molecules is more restricted in malignant tumors due to a high cellular density associated with numerous intact cell membranes [2]. Consequently, malignant tumors typically demonstrate hyperintensity due to their high cellularity on DWI. Meanwhile, the quantification of this diffusion can be achieved by calculating the apparent diffusion coefficient (ADC). Recently, the clinical utility of DWI in the routine management of gynecological malignancies has been well-established. In endometrial cancer, DWI offers an equivalent diagnostic performance comparable to that of dynamic contrast-enhanced MRI (DCE-MRI) for assessing deep myometrial invasion [3]. In cervical cancer, the combination of DWI with T2WI improves specificity for evaluating parametrial invasion at initial staging and for detecting residual tumors after concurrent chemoradiotherapy (CCRT) [4]. In uterine sarcoma, DWI is also an important component of multiparametric MRI, contributing to the differentiation of sarcomas from benign leiomyomas [5].

Single-shot echo planar imaging (ss-EPI) is the most commonly used acquisition technique for DWI. In ss-EPI, the sequence acquires the entire k-space with a single radiofrequency excitation. This sequence is relatively insensitive to the influence of macroscopic patient motion because of a very short scan time [6]. Accordingly, ss-EPI is highly practical for routine clinical DWI of various body regions, including the pelvis. However, ss-EPI has limitations such as geometric distortion and image blurring [7]. Geometric distortion arises from B0 field inhomogeneities or magnetic susceptibility effects. Because ss-EPI sequences have a relatively long readout time, these field variations can cause an accumulation of phase errors. These errors lead to spatial misregistration of the signal along the phase-encode direction, causing the geometrical distortion of the images. This is particularly problematic in pelvic MRI due to the complex magnetic susceptibility environment created by air-tissue interfaces of the bowel and vagina [8]. Furthermore, image blurring caused by tissue T2* decay contributes to the low spatial resolution of EPI.

Multi-shot echo planar imaging (ms-EPI) addresses the limitations of ss-EPI by partitioning k-space acquisition across multiple shots [9]. This approach shortens the per-shot echo-train length (in interleaved ms-EPI) or the echo spacing (in readout-segmented EPI), which significantly reduces geometric distortion and image blurring [7]. MUltiplexed Sensitivity Encoding (MUSE) is a notable interleaved ms-EPI technique that partitions k-space sampling along the phase-encoding direction. A key feature of MUSE is its ability to inherently and reliably correct nonlinear phase variations that occur between shots without requiring navigator echoes [10]. This capability effectively suppresses the ghosting artifacts that would otherwise arise from inter-shot phase inconsistencies. Recent studies suggest that DWI acquired with MUSE demonstrated significantly better image quality in uterine MRI [11, 12]. Given its reported utility in uterine MRI as an advanced EPI-based DWI technique, MUSE-DWI provides a clinically relevant reference for evaluating newer DWI techniques in this field.

Periodically Rotated Overlapping ParallEL Lines with Enhanced Reconstruction (PROPELLER) sequence is a fast/turbo spin echo (FSE/TSE)-based technique that intrinsically avoids geometric distortion. This sequence is commercially branded as “PROPELLER” by GE Healthcare and “BLADE” by Siemens Healthineers and is used in clinical practice. This technique acquires a rectangular strip, or “blade”, during each spin-echo train. Multiple rotated blades are then acquired over several shots to fully sample a circular k-space region [13]. It is robust to patient motion because the data for each blade can be analyzed to correct for object motion by oversampling the central k-space. Furthermore, it is relatively insensitive to B0-related artifacts [14]. At present, PROPELLER-DWI has been applied in brain [15–18], orbit [19, 20], middle ear [21, 22], paranasal sinuses [23], prostate [24] and rectum [25]. Despite these advantages, conventional PROPELLER-DWI has been limited by relatively long scan times and reduced SNR. The application of deep learning reconstruction (DLR) to PROPELLER-DWI may help overcome these limitations by improving image noise and enabling clinically feasible acquisition. Therefore, DLR-applied PROPELLER-DWI may represent a more future-oriented imaging package for MRI of uterine malignancies, compared with MUSE-DWI, for which DLR is currently unavailable in our clinical implementation. However, to our knowledge, the clinical efficacy of DLR-applied PROPELLER-DWI in uterine malignancies has not yet been reported. The purpose of this study was to compare DLR-applied PROPELLER-DWI and MUSE-DWI in uterine malignancies, and to investigate their subjective and objective image quality and apparent diffusion coefficient (ADC) values.

## Materials and methods

### Patient population

This single-center retrospective study protocol was approved by the institutional ethics committee (24A034) and the requirement for written informed consent was waived. Between October 2023 and May 2024, 158 MRI examinations were performed for the evaluation of uterine lesions at our institution. Among these, 109 examinations were performed for uterine malignancies, including cervical cancer, endometrial cancer, and endometrial stromal sarcoma, whereas 49 examinations were performed for benign uterine conditions. During the study period, both MUSE-DWI and DLR-applied PROPELLER-DWI were incorporated into our MRI protocol in patients with uterine malignancies. However, because acquisition of both sequences depended on scanner availability and examination workflow in routine clinical practice, they were not obtained in all examinations. Consequently, 43 examinations lacking these DWI sequences were excluded from the analysis. Thus, 66 examinations in which both MUSE-DWI and DLR-applied PROPELLER-DWI were acquired were included in this study. The final cohort included 66 MRI examinations performed in 48 female patients. The mean age of the patients was 59.0 years (range: 30–86 years). The clinical indications were as follows: initial staging of cervical cancer (n = 11), during or after CCRT of cervical cancer (n = 37), after chemotherapy of cervical cancer (n = 2), initial staging of endometrial cancer (n = 12), after chemotherapy of endometrial cancer (n = 1), post-operative recurrence of endometrial cancer (n = 2), and high-grade endometrial stromal sarcoma (n = 1). The inclusion criterion was that patients had undergone both MUSE-DWI and DLR-applied PROPELLER-DWI.

### MRI technique

All MRI examinations were acquired on a 3 T MRI (Signa Architect, GE Healthcare, Milwaukee, WI, USA) with 30-channel air anterior array (AA) coil and 40-channel spine posterior array (PA) coil. No patients received an antispasmodic agent to reduce bowel motion prior to image acquisition. All patients were scanned in the supine position and instructed to breathe freely during the scan.

The imaging sequences used for evaluation in this study were performed with the scanning parameters summarized in Table 1. T2WI were acquired in three planes. Axial T2WI was acquired using an FSE sequence. Coronal T2WI employed a single-shot (ssh) technique. Sagittal T2WI was acquired using either a ssh technique or a PROPELLER technique. Notably, the sagittal plane acquisition was performed obliquely to the long axis of the uterus (oblique sagittal). MUSE-DWI and PROPELLER-DWI sequences were acquired in the oblique sagittal plane using b-values of 0 and 800 s/mm^2^. Specifically, MUSE-DWI was performed with two shots and an acceleration factor of 2. For the PROPELLER-DWI sequence, an echo train length of 24 was employed. Commercially available DLR technology (AIR Recon DL, GE Healthcare, Milwaukee, WI, USA) was used for PROPELLER-DWI sequences. The ADC map for each DWI sequence was automatically calculated using a mono-exponential fitting of two different b-values.

**Table 1:** Summary of MRI scanning parameters

Sequences	Axial T2WI	Coronal T2WI	Oblique sagittal T2WI		MUSE-DWI	PROPELLER-DWI
			ssh-T2WI	PROPELLER-T2WI		
TR/TE(ms)	4500/103	2000/172	2000/130	5477/121	3212/72	3635/69
FOV(mm)	280 × 280	350 × 350	250 × 250	250 × 250	280 × 280	280 × 280
Matrix size	384 × 356	320 × 272	228 × 224	388 × 388	112 × 166	128 × 128
Slice thickness (mm)	5	5	3	3	3.6	3.6
Slice spacing (mm)	1.5	1	0.6	0.6	0.6	0.6
Flip angle (°)	120	130	130	111	90	110
Band width (kHz)	91	83	83	100	250	83
Number of excitations	1	1	1	1.5	9	3
Scan time (s)	104	50	40	125	193	217

TE indicates the effective TE for fast/turbo spin echo (FSE/TSE)-based sequences (ssh-T2WI, and PROPELLER sequences)

*TR/TE* Repetition time/echo tim, *FOV* field of view, *T2WI* T2-weighted imaging, *ssh-T2WI* single-shot T2WI, *PROPELLER* Periodically Rotated Overlapping ParallEL Lines with Enhanced Reconstruction, *MUSE* MUltiplexed Sensitivity Encoding, *DWI* diffusion-weighted imaging

### Qualitative image analysis

All images were transferred to picture archiving and communication system (PACS) workstation monitors (EV Report, PSP corporation, Tokyo, Japan). Two independent radiologists performed the subjective image quality assessment (reader 1 with 6 years and reader 2 with 24 years of experience in diagnostic radiology, respectively). The readers were blinded to the type of DWI sequences and all patient clinical information. For lesion assessment, the readers were allowed to refer to the corresponding T2-weighted images in three planes as the anatomical reference standard for identifying the target lesion. The qualitative visual assessment was performed on the b = 800 s/mm^2^ images using a 5-point Likert scale based on the following criteria: overall image quality, artifacts, sharpness and lesion conspicuity (Table 2). Sharpness was defined as the clarity of the uterine boundaries in patients without a history of hysterectomy. In patients with an identifiable target lesion, lesion conspicuity was also assessed. For lesion conspicuity assessment, an unidentifiable lesion was defined as a lesion conspicuity score of 1 on the 5-point Likert scale. If the two readers disagreed on the identifiability of a lesion, a consensus decision was reached through joint review.

**Table 2:** Image assessment based on the 5-point Likert scale

Score	Overall image quality	Artifacts	Sharpness	Lesion conspicuity
1	Non-diagnostic	Non-diagnostic	Non-diagnostic	Lesion unidentifiable
2	Substantial deficits in image quality	Substantial impact on diagnosis	Not sharp	No differentiation between lesion and normal anatomy
3	Moderate image quality	Moderate impact on diagnosis	A little sharp	Subtle lesion with poorly defined edges
4	Good image quality	Little impact on image diagnosis	Moderately sharp	Well-seen lesion with poorly defined edges
5	Excellent image quality	No artifact	Satisfying sharp	Well-seen lesion with well-defined edges

### Quantitative image analysis

Quantitative measurements were performed after completion of the qualitative assessment. The mean ADC values were measured independently by the two readers. Apparent signal-to-noise ratio (aSNR) and apparent contrast-to-noise ratio (aCNR) were measured by reader 1 only. These quantitative evaluations were restricted to cases where both readers identified the tumor as clearly visible during the qualitative assessment. Cases in which the tumor was too small to allow reliable region of interest (ROI) placement were consequently excluded from all quantitative analysis.

**Fig. 1:**
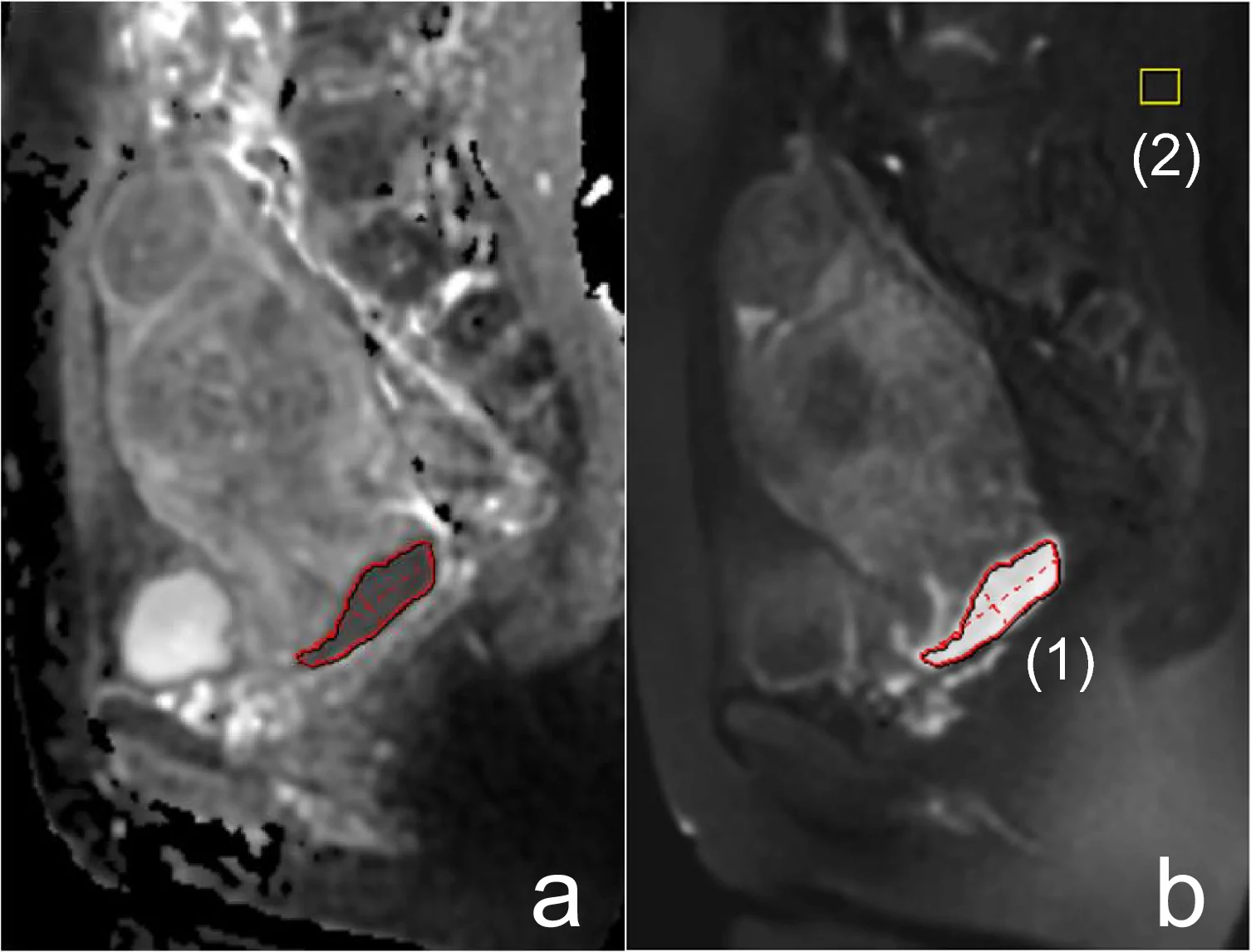
ROI placement for quantitative measurements on diffusion-weighted imaging (DWI) and apparent diffusion coefficient (ADC) maps. **a** A manual ROI placed on the tumor on the ADC map derived from Periodically Rotated Overlapping ParallEL Lines with Enhanced Reconstruction (PROPELLER)-DWI. **b** A manual ROI and a square ROI placed on (1) the tumor and (2) the erector spinae muscle on b = 800 s/mm^2^ PROPELLER-DWI, respectively

The mean ADC values were calculated by drawing operator-defined ROIs within the tumor on the ADC maps derived from both MUSE-DWI and DLR-applied PROPELLER-DWI sequences (Fig. 1). ROIs were placed only within the solid and homogeneous components of the tumor, specifically avoiding cystic, necrotic, or inhomogeneous areas. The ROIs were made as large as possible while ensuring the placement at near-identical sites on both sequences. To facilitate accurate placement, readers were permitted to refer to the corresponding T2WI.

The same ROIs used for ADC measurements were then copied and pasted onto the b = 800 s/mm^2^ images (Fig. 1). Because DLR alters image noise characteristics nonlinearly, the SNR and CNR measurements in this study were treated as aSNR and aCNR, rather than true physical noise metrics. The average signal value within the tumor ROI was denoted as S_tumor_. The aSNR was defined as S_tumor_ divided by the standard deviation (SD) of the tumor signal (SD_tumor_):

$$ {\mathrm{aSNR}} = {\mathrm{S}}_{{{\mathrm{tumor}}}} /{\mathrm{SD}}_{{{\mathrm{tumor}}}} $$

To calculate the aCNR, an additional ROI was placed on a muscle with relatively homogeneous signal intensity (Fig. 1). Specifically, either the erector spinae muscle or the rectus abdominis muscle was selected according to which had the greater anterior–posterior diameter. The average signal value within this muscle ROI was denoted as S_muscle_. The aCNR was then defined as the difference between S_tumor_ and S_muscle_ divided by the standard deviation of the muscle signal (SD_muscle_):

$$ {\mathrm{aCNR}} = \left| {{\mathrm{S}}_{{{\mathrm{tumor}}}} - {\text{ S}}_{{{\mathrm{muscle}}}} } \right|/{\mathrm{SD}}_{{{\mathrm{muscle}}}} $$

### Statistical analysis

Inter-reader agreement for the qualitative image quality scores was assessed using the weighted kappa (*κ*) statistic. Agreement levels were categorized as follows: poor (< 0), slight (0.01–0.20), fair (0.21–0.40), moderate (0.41–0.60), good (0.61–0.80), and excellent (0.81–0.99). Additionally, assessments with slight inter-reader agreement were further evaluated using contingency tables to investigate the patterns of disagreement that contributed to the low κ values. To assess significant differences in both qualitative and quantitative parameters between the MUSE-DWI and DLR-applied PROPELLER-DWI groups, the Wilcoxon signed-rank test was employed. Statistical analyses were performed using IBM SPSS Statistics version 29.0 (IBM, Armonk, NY, USA). A *P*-value of < 0.05 was considered statistically significant.

## Results

The flow of examinations included in each analysis is shown in Supplementary Fig. S1. The number of examinations differed among analyses according to the evaluability of each parameter.

### Inter-reader agreement

The statistical results regarding inter-reader agreement are presented in Table 3. Assessment of sharpness was conducted on 64 cases, excluding the two patients who had undergone a hysterectomy. Regarding lesion conspicuity, the analysis was restricted to 32 cases where the lesion was identified by both readers, independently and concordantly. Although the degree of agreement ranged from slight to moderate for most parameters, a strong level of agreement (*κ* = 0.696, good) was specifically achieved for lesion conspicuity assessed on DLR-applied PROPELLER-DWI. To further examine the parameters with slight inter-reader agreement, reader 1 × reader 2 contingency tables were generated for artifacts on DLR-applied PROPELLER-DWI and sharpness on both MUSE-DWI and DLR-applied PROPELLER-DWI (Supplementary Tables S1–S3). For artifact assessment on DLR-applied PROPELLER-DWI, complete agreement was observed in 35 of 66 examinations (53.0%), and disagreement by only one score was observed in 30 of 66 examinations (45.5%). Additionally, most disagreements occurred between scores of 4 and 5. For sharpness assessments on both sequences, most disagreements similarly occurred between scores of 4 and 5.

**Table 3:** Inter-reader agreement

	Overall image quality	Artifacts	Sharpness	Lesion conspicuity
N	66	66	64	32
MUSE-DWI	0.358	0.396	0.072	0.434
PROPELLER-DWI	0.292	0.048	0.200	0.696

Data are κ values

Agreement levels: poor (< 0), slight (0.01–0.20), fair (0.21–0.40), moderate (0.41–0.60), good (0.61–0.80), and excellent (0.81–0.99)

*MUSE* MUltiplexed Sensitivity Encoding, *DWI* diffusion-weighted imaging, *PROPELLER* Periodically Rotated Overlapping ParallEL Lines with Enhanced Reconstruction

### Qualitative image analysis

**Fig. 2:**
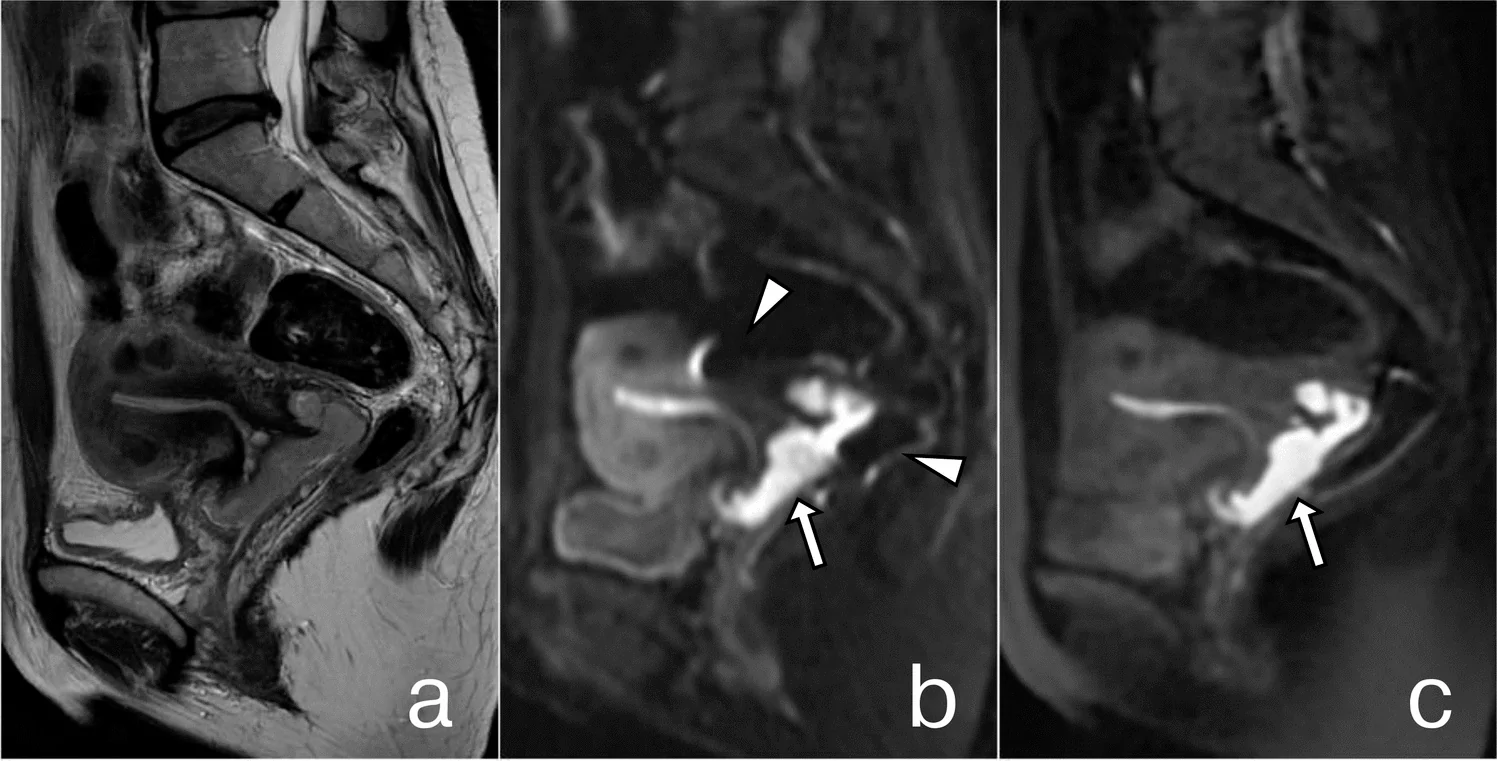
Initial staging of cervical cancer in a 42-year-old patient. **a** T2-weighted image. **b** MUltiplexed Sensitivity Encoding (MUSE) diffusion-weighted imaging (DWI). **c** Periodically Rotated Overlapping ParallEL Lines with Enhanced Reconstruction (PROPELLER)-DWI. PROPELLER-DWI demonstrates fewer artifacts (arrowheads), clearer lesion conspicuity (arrows) and better image quality, compared to MUSE-DWI

**Fig. 3:**
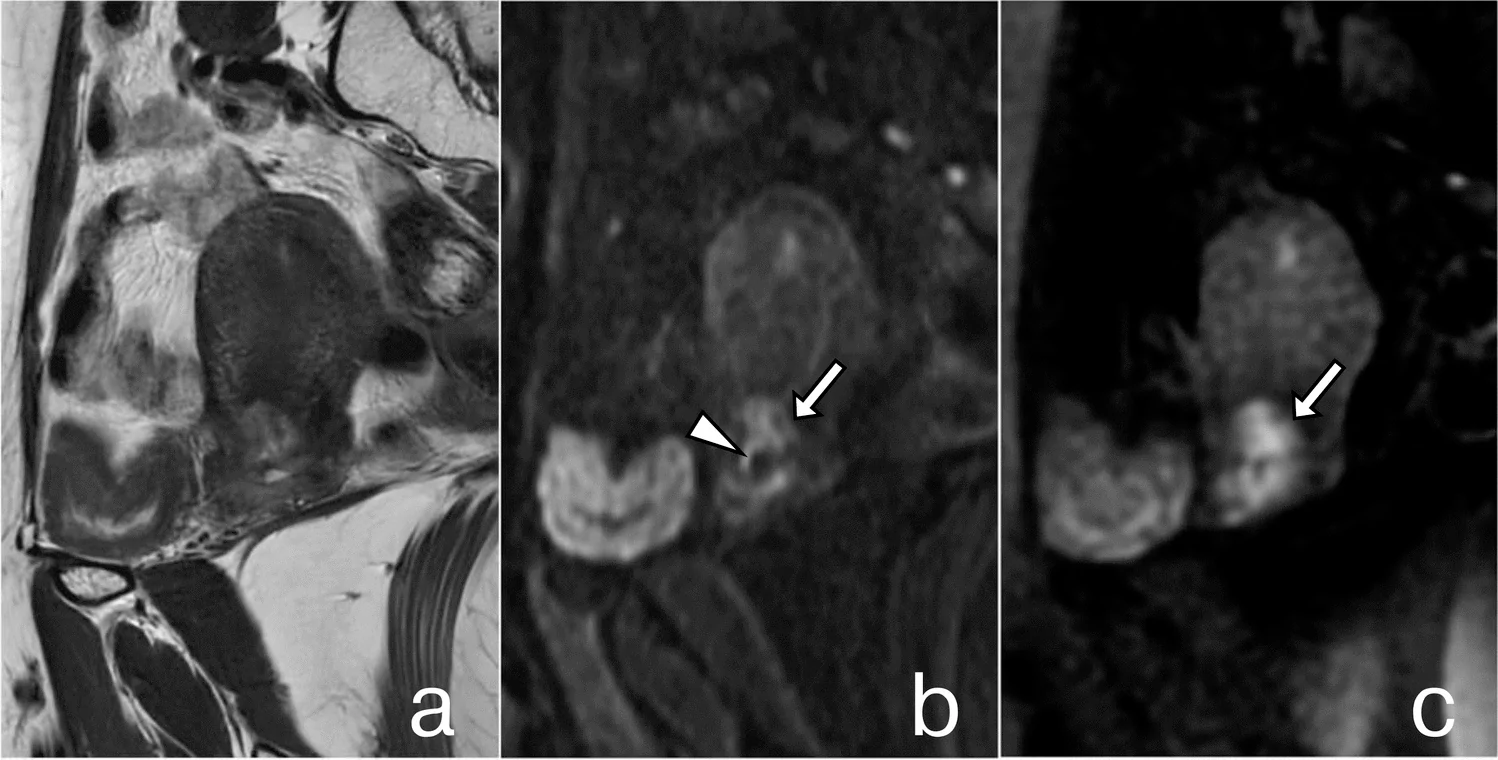
Assessment of treatment response after concurrent chemoradiotherapy (CCRT) for cervical cancer in a 35-year-old patient. **a** T2-weighted image. **b** MUltiplexed Sensitivity Encoding (MUSE) diffusion-weighted imaging (DWI). **c** Periodically Rotated Overlapping ParallEL Lines with Enhanced Reconstruction (PROPELLER)-DWI. PROPELLER-DWI reduces intravaginal gas-induced artifacts (arrowheads), improving residual tumor visualization (arrows) compared to MUSE-DWI

Qualitative assessment results are summarized in Table 4. The analysis of lesion conspicuity was conducted on 39 cases where the two readers reached a consensus that the lesion was identifiable. DLR-applied PROPELLER-DWI demonstrated significantly fewer artifacts (Reader 1: *p* < 0.001; Reader 2: *p* < 0.001) and better lesion conspicuity (Reader 1: *p* < 0.001; Reader 2: *p* = 0.012) compared to MUSE-DWI (Figs. 2 and 3). Conversely, there was no statistically significant difference between the two sequences regarding overall image quality and sharpness.

**Table 4:** Qualitative image analysis

	Overall image quality	Artifacts	Sharpness	Lesion conspicuity
N	66	66	64	39
Reader 1				
MUSE-DWI	4.36	3.94	4.23	4.10
PROPELLER-DWI	4.18	4.48	4.13	4.62
* p* value	0.062	< 0.001	0.312	< 0.001
Reader 2				
MUSE-DWI	4.02	3.74	4.78	4.44
PROPELLER-DWI	4.20	4.85	4.64	4.69
* p* value	0.070	< 0.001	0.072	0.012

Data are mean

*MUSE* MUltiplexed Sensitivity Encoding, *DWI* diffusion-weighted imaging, *PROPELLER* Periodically Rotated Overlapping ParallEL Lines with Enhanced Reconstruction

### Quantitative image analysis

The results of the quantitative assessment are presented in Table 5. The analysis was conducted in 36 cases because three cases were excluded due to lesions that were too small for reliable measurement. The mean ADC values were significantly higher in DLR-applied PROPELLER-DWI compared to MUSE-DWI for both readers (*p* < 0.001 for both). Furthermore, aSNR was significantly higher in DLR-applied PROPELLER-DWI compared to MUSE-DWI (*p* < 0.001). Similarly, aCNR was significantly higher with DLR-applied PROPELLER-DWI than with MUSE-DWI (*p* < 0.001).

**Table 5:** Quantitative image analysis

N = 36	ADC value (× 10^−3^mm^2^/s)		aSNR	aCNR
	Reader 1	Reader 2		
MUSE-DWI	1.10 ± 0.36	1.13 ± 0.37	7.73 ± 2.95	22.79 ± 17.01
PROPELLER-DWI	1.41 ± 0.40	1.42 ± 0.39	9.63 ± 3.44	39.92 ± 25.47
*p* value	< 0.001	< 0.001	< 0.001	< 0.001

Data are mean ± standard deviation

*MUSE* MUltiplexed Sensitivity Encoding, *DWI* Diffusion-weighted imaging, *PROPELLER* Periodically Rotated Overlapping ParallEL Lines with Enhanced Reconstruction, *ADC* Apparent diffusion coefficient; *aSNR* apparent signal-to-noise ratio, *aCNR* apparent contrast-to-noise ratio

## Discussion

Our results demonstrated that DLR-applied PROPELLER-DWI had significantly fewer artifacts, better lesion conspicuity, and higher aSNR and aCNR compared to MUSE-DWI in uterine malignancies. We also observed that no significant difference was detected in overall image quality or sharpness between the two sequences. Collectively, these findings suggest that the utilization of DLR-applied PROPELLER-DWI holds the potential to elevate diagnostic confidence in uterine malignancies. To our knowledge, this is the first investigation to evaluate DLR-applied PROPELLER-DWI against a ms-EPI technique such as MUSE in the assessment of uterine malignancies.

PROPELLER-DWI is intrinsically robust against motion and susceptibility-related artifacts [13, 14]. Our results suggest that DLR-applied PROPELLER-DWI offers further advantages in artifact suppression and lesion visualization compared to MUSE-DWI. A previous study has demonstrated that MUSE-DWI, compared with conventional ss-EPI, reduces distortion and artifacts, improves image sharpness and SNR, and enhances lesion conspicuity [11]. Several studies have reported comparisons between PROPELLER-DWI and the readout-segmented EPI (rs-EPI) technique, which is another type of ms-EPI sequence, similar to MUSE-DWI. These previous reports consistently found that PROPELLER-DWI yielded fewer geometric distortion and susceptibility artifacts compared to rs-EPI [16–20, 22, 23]. Furthermore, PROPELLER-DWI showed superior lesion conspicuity [16, 17, 19, 23] and higher CNR [22, 23]. These results are consistent with the findings of the current study.

The clinical adoption of the PROPELLER technique has historically been limited. However, the combination of PROPELLER and DLR now allows for its feasible implementation in clinical practice. DLR is an emerging technology utilizing deep convolutional neural networks based on supervised learning. By training on pairs of noisy input images and high SNR ground truth images, DLR effectively mitigates noise and enhances overall image quality, leading to generate images with higher SNR. Furthermore, when integrated with accelerated acquisition techniques such as parallel imaging or compressed sensing, DLR facilitates significant reductions in scan times while preserving or even enhancing diagnostic image quality [26]. While PROPELLER techniques traditionally suffered from drawbacks such as prolonged scan time [27] and potential SNR reduction [22, 23, 28], the application of DLR allows for the mitigation of these inherent limitations. Specifically, several reports have demonstrated that the combination of PROPELLER and DLR leads to improved SNR [29, 30]. Furthermore, Matsumoto et al. [25] reported that PROPELLER-DWI with DLR yielded a significantly higher SNR in rectal MRI when compared to conventional DWI with DLR. Consistent with these reports, our findings demonstrate that the utilization of DLR with PROPELLER-DWI enables to improve aSNR and lesion conspicuity at a clinically feasible scan time for routine practice. Although the scan time of DLR-applied PROPELLER-DWI was slightly longer than that of MUSE-DWI in our protocol (217 s vs. 193 s), the additional scan time was limited and was acceptable in our routine clinical workflow, particularly considering the improved artifact reduction and lesion conspicuity. Therefore, DLR-applied PROPELLER-DWI in uterine malignancies is expected to improve the clinical utility such as the detection of small lesions, the accurate local staging, and the early diagnosis of post-treatment recurrence. However, because the present study did not include diagnostic-performance endpoints, the diagnostic value of DLR-applied PROPELLER-DWI for uterine malignancies should be evaluated in future studies.

Importantly, ADC values measured on DLR-applied PROPELLER-DWI were significantly higher than those obtained with MUSE-DWI, indicating that the choice of diffusion sequence can influence quantitative diffusion parameters. Conflicting results have been reported in the literature regarding differences in ADC values between PROPELLER-DWI and rs-EPI [17–20, 22, 23]. In particular, Okuchi et al. [18] reported that the direction of ADC differences varied depending on the anatomical region. These differences may also be related to several acquisition-related factors, including differences in the severity of susceptibility artifacts and geometric distortion, as well as sequence-specific parameters such as the timing of motion probing gradient application and TR [18, 20, 28]. Additionally, a phantom study using radial acquisition regime DWI (RADAR-DWI), a sequence utilizing radial k-space sampling similar to PROPELLER, reported higher ADC values than conventional EPI-DWI, suggesting that a mixture of different TEs in the low-frequency part of k-space may affect ADC measurements [31]. Thus, several factors contribute to differences in ADC values between PROPELLER-DWI and EPI-based DWI. Beyond acquisition-related factors, DLR itself may also affect ADC values, although its reported effects have been inconsistent in previous studies [32–34]. Therefore, the higher ADC values observed with DLR-applied PROPELLER-DWI in the present study may partly reflect the combined effect of PROPELLER acquisition and DLR, rather than PROPELLER acquisition alone. This sequence-dependent difference in ADC values has important clinical implications. Because established ADC cut-off values for gynecologic tumors have largely been derived from conventional EPI-based DWI, they may not be directly applicable to DLR-applied PROPELLER-DWI. In addition, because conventional ss-EPI was not included in the present study, we could not determine whether ADC values obtained with MUSE-DWI were equivalent to those obtained with conventional ss-EPI. Further studies are needed to establish sequence-specific ADC interpretation.

In our study, inter-reader agreement for lesion conspicuity on DLR-applied PROPELLER-DWI reached a good level, indicating that readers were more consistent when specifically judging the visibility of the lesion. Conversely, inter-reader agreement for other qualitative parameters ranged only from slight to moderate. Several factors may account for this finding. First, overall image quality is inherently difficult to define and is influenced by individual reader preference, which may have introduced greater subjectivity and variability in scoring [35]. Second, although artifacts were graded based on their interference with diagnostic evaluation, the assessment remained partly subjective, and reader experience may have contributed to differences in the thresholds for judging the impact of minor residual artifacts on diagnostic confidence [36]. Third, the assessment of sharpness can be challenging in uterine MRI because regions with distinct uterine contours and areas with blurred margins often coexist within the same examination. This coexistence makes it difficult for readers to integrate these mixed features into a single score. Indeed, inter-reader agreement is an important imaging interpretation-related outcome in radiological research, and κ statistics are commonly used for categorical or ordinal assessments [37]. However, the contingency tables for parameters with slight agreement demonstrated that most disagreements occurred within the favorable-score range. Consequently, the low κ values are considered to reflect reader-dependent thresholds and high-score skewness, rather than substantial disagreement regarding diagnostic acceptability [38].

Our study has several limitations. First, this was a single-center, retrospective study with a relatively small sample size. This characteristic may limit the generalizability of our findings. Second, the cohort was restricted to uterine malignancies, including cervical cancer, endometrial cancer, and endometrial stromal sarcoma. Benign uterine conditions, such as leiomyomas and adenomyosis, as well as ovarian and adnexal diseases, were not included. Therefore, our findings may not be generalizable to routine all-inclusive pelvic MRI or to benign uterine and adnexal conditions. Third, even within uterine malignancies, the cohort included heterogeneous diseases and imaging time points, including both pre- and post-treatment examinations. This heterogeneity underscores the need for future large-scale cohort studies to perform detailed subgroup analyses. Fourth, the quantitative evaluation of ADC, aSNR, and aCNR was limited to cases with well-visualized tumors that allowed reliable ROI placement. Therefore, these findings may not extend to less well-visualized lesions. Fifth, we did not separately record the frequency or severity of specific artifact types, such as geometric distortion, ghosting, motion artifacts, or blurring. Therefore, the present study cannot determine which specific artifact subtype contributed most to the observed differences between the sequences. Sixth, DLR was applied to PROPELLER-DWI but not to MUSE-DWI because DLR was not available for MUSE-DWI in our current clinical implementation. This introduces a confounding factor, making it difficult to isolate whether the observed improvements in aSNR and aCNR are attributable to PROPELLER acquisition or DLR. Furthermore, DLR changes image noise statistics nonlinearly. Therefore, aSNR and aCNR values based on the SD within a single ROI should be interpreted cautiously and should not be regarded as true physical noise metrics. Finally, complete blinding was difficult to achieve because experienced radiologists may recognize the characteristic appearances of MUSE-DWI and DLR-applied PROPELLER-DWI, including differences in image texture and matrix size. If readers recognized the sequences, this may have introduced identification bias, potentially favoring DLR-applied PROPELLER-DWI in subjective assessments such as artifacts and lesion conspicuity.

In conclusion, DLR-applied PROPELLER-DWI provided high-quality images for uterine malignancies, demonstrating fewer artifacts, better lesion conspicuity, and higher aSNR and aCNR than MUSE-DWI, although it yielded higher ADC values. These findings suggest that DLR-applied PROPELLER-DWI may be a promising alternative to conventional DWI in uterine malignancies.

## Supplementary Information

Below is the link to the electronic supplementary material.

Supplementary file1 (PPTX 36 KB) **Supplementary Fig. S1** Flow diagram of examinations included in each analysis. Overall image quality and artifacts were assessed in all 66 examinations. Sharpness was assessed in 64 examinations after excluding two post-hysterectomy cases. Lesion conspicuity was evaluated in 39 examinations after consensus review. Inter-reader agreement for lesion conspicuity was calculated in 32 examinations in which both readers independently identified the lesion. Quantitative analysis was performed in 36 examinations after excluding three lesions that were too small for reliable ROI placement.

Supplementary file2 (DOCX 15 KB)
