# Supplementary material for: Deep learning reconstruction-applied PROPELLER diffusion-weighted imaging in uterine malignancies: a comparison study with MUSE
Source: Jpn J Radiol. 2026 Jul 15;44(11):1992–2001. doi: 10.1007/s11604-026-02048-9 (PMC13722724; doi:10.1007/s11604-026-02048-9)
Supplement: Supplementary file 1 [file 11604_2026_2048_MOESM2_ESM.docx]

Supplementary Table S1 Reader 1 × Reader 2 contingency table for artifact scores on DLR-applied PROPELLER-DWI

| Reader 1 score / Reader 2 score | 1 | 2 | 3 | 4 | 5 | Total |
| --- | --- | --- | --- | --- | --- | --- |
| 1 | 0 | 0 | 0 | 0 | 0 | 0 |
| 2 | 0 | 0 | 0 | 0 | 0 | 0 |
| 3 | 0 | 0 | 0 | 0 | 1 | 1 |
| 4 | 0 | 0 | 0 | 6 | 26 | 32 |
| 5 | 0 | 0 | 0 | 4 | 29 | 33 |
| Total | 0 | 0 | 0 | 10 | 56 | 66 |

Data are numbers of examinations. Rows and columns correspond to artifact scores assigned by reader 1 and reader 2, respectively.

Supplementary Table S2 Reader 1 × Reader 2 contingency table for sharpness scores on MUSE-DWI

| Reader 1 score / Reader 2 score | 1 | 2 | 3 | 4 | 5 | Total |
| --- | --- | --- | --- | --- | --- | --- |
| 1 | 0 | 0 | 0 | 0 | 0 | 0 |
| 2 | 0 | 0 | 0 | 0 | 1 | 1 |
| 3 | 0 | 0 | 0 | 1 | 6 | 7 |
| 4 | 0 | 0 | 1 | 9 | 22 | 32 |
| 5 | 0 | 0 | 0 | 2 | 22 | 24 |
| Total | 0 | 0 | 1 | 12 | 51 | 64 |

Data are numbers of examinations. Rows and columns correspond to sharpness scores assigned by reader 1 and reader 2, respectively.

Supplementary Table S3 Reader 1 × Reader 2 contingency table for sharpness scores on DLR-applied PROPELLER-DWI

| Reader 1 score / Reader 2 score | 1 | 2 | 3 | 4 | 5 | Total |
| --- | --- | --- | --- | --- | --- | --- |
| 1 | 0 | 0 | 0 | 0 | 0 | 0 |
| 2 | 0 | 0 | 0 | 0 | 0 | 0 |
| 3 | 0 | 0 | 2 | 4 | 6 | 12 |
| 4 | 0 | 0 | 0 | 11 | 21 | 32 |
| 5 | 0 | 0 | 1 | 2 | 17 | 20 |
| Total | 0 | 0 | 3 | 17 | 44 | 64 |

Data are numbers of examinations. Rows and columns correspond to sharpness scores assigned by reader 1 and reader 2, respectively.
